# Surface Inspection and Description in Metrology and Tribology—Vol. 2

**DOI:** 10.3390/ma19010071

**Published:** 2025-12-24

**Authors:** Michal Wieczorowski, Maxence Bigerelle, Christopher A. Brown, Pawel Pawlus, Rafal Reizer, Alejandro Pereira

**Affiliations:** 1 Division of Metrology and Measurement Systems, Faculty of Mechanical Engineering, Institute of Mechanical Technology, Poznan University of Technology, Piotrowo Street 3, 60-965 Poznan, Poland; 2 Laboratoire d’Automatique, de Mécanique et d’Informatique Industrielles et Humaines, Lamih, Université Polytechnique Hauts-de-France, UMR CNRS 8201, 59300 Valenciennes, France; maxence.bigerelle@univ-valenciennes.fr; 3 Mechanical and Materials Engineering Department, Worcester Polytechnic Institute, Worcester, MA 01609, USA; brown@wpi.edu; 4 Faculty of Mechanical Engineering and Aeronautics, Rzeszow University of Technology, Powstancow Warszawy 8 Street, 35-959 Rzeszow, Poland; ppawlus@prz.edu.pl; 5 Faculty of Exact and Technical Sciences, Institute of Materials Engineering, University of Rzeszow, Pigonia Street 1, 35-310 Rzeszow, Poland; rreizer@ur.edu.pl; 6 Manufacturing Engineering Group (GEF) EEI Campus Lagoas, University of Vigo, 36310 Vigo, Spain; apereira@uvigo.es

## 1. Introduction

As was the case with the first volume, this Special Issue aims at providing an overview of global developments in surface description and inspection from the perspectives of metrology and tribology. Although many years have passed since the first studies on surface topography [1,2], and several books have since been published on the subject [3,4,5], this area not only remains relevant but is also gaining increasing popularity. This is because surfaces play a fundamental role: every physical object in the human environment is bounded by a surface. When considered as an interface between solid, liquid, or gaseous phases, the surface becomes a site of intense exchange of energy and matter. Surfaces determine a wide range of phenomena, including resistance to wear and corrosion, thermal and electrical conductivity, wettability, adhesion, and tribological behavior. And tribology, as a discipline, focuses specifically on the study of friction, wear, and lubrication of surfaces in relative motion.

The topography of both contacting surfaces determines the actual area of contact between two bodies, as real contact occurs only at the asperity summits. The greater the surface roughness, the smaller the true contact area and the higher the local contact pressures at individual asperities. This, in turn, may lead to more intensive abrasive and adhesive wear. Consequently, the actual contact area constitutes only a small fraction of the nominal (apparent) contact area. From a tribological perspective, surface topography governs several key factors: the number and distribution of real contact spots, the pressure distribution across the contact area, the retention and transport of the lubricating medium, and the formation of wear layers or tribofilms. These microstructural features critically influence frictional behavior and wear mechanisms during the operation of tribological systems.

## 2. Metrological Approach to Surfaces

Through surface engineering techniques—such as machining, texturing, hardening, and coatings [6]—we have learned to control surface properties and effectively influence tribological phenomena. This includes reducing friction in situations where energy losses are undesirable, or increasing it when higher traction is required [7]. Various methods have been developed to generate specific surface textures tailored for different functional applications [8,9,10,11]. We are now capable of minimizing wear through surface hardening and lubrication, and of preventing adhesion by applying specialized coatings or incorporating surface-modifying additives. Deliberate surface design and optimization have become increasingly important across nearly all industrial sectors, ranging from large-scale wind turbine gearboxes to microscale MEMS accelerometers, and from railway bearings to hip joint implants. Moreover, the analysis of wear debris, including its size, morphology, and chemical composition, offers diagnostic insights into the underlying wear mechanisms [12].

Given the aspects discussed above, surface roughness metrology continues to evolve, seeking new and improved methods for characterizing and describing surface irregularities [13], as well as advanced techniques for surface inspection and digitization [14,15,16]. An important research focus concerns the separation of various surface roughness components, especially at the microscale, which is closely tied to filtering techniques and multiscale analysis [17,18]. These approaches allow surface microtopography to be decomposed into distinct spatial scales, providing deeper insight into the relationship between microtopographic features and functional properties such as friction, wear, lubrication behavior, adhesion, and fatigue performance. From an industrial perspective, these developments align with the emergence of Metrology 4.0, which represents the measurement-oriented facet of Industry 4.0 within a digital and information-driven ecosystem [19,20]. In this context, surface microtopography measurement systems are no longer standalone tools but integral elements of connected manufacturing environments. High-resolution microtopographic data are increasingly integrated with digital twins, CAQ (Computer-Aided Quality) systems, augmented reality technologies, and artificial intelligence. Measurement data are analyzed in real time and used for the automatic adjustment of manufacturing process parameters, enabling adaptive and self-optimizing production environments in which surface microtopography becomes a key control variable. Looking ahead, visionary concepts associated with Industry 5.0 suggest that surfaces—and, in particular, their microtopographic structure—will continue to play a critical functional role in successive industrial revolutions. Importantly, research related to surface microtopography and the information it contains extends far beyond classical mechanical or chemical engineering, encompassing disciplines such as data science, applied mathematics, computer vision, and artificial intelligence. Relevant studies can now be found across diverse scientific disciplines, including civil engineering [21,22], bioengineering [23,24], sports science, and even archeology [25,26]. This broad interest highlights the interdisciplinary nature and growing relevance of surface science in both applied and fundamental research contexts.

In engineering metrology, the surface of an object represents a fundamental subject of measurement and analysis. A wide range of 2D and 3D parameters are used for their quantitative characterization [27,28]. These parameters have been standardized and are available both in general-purpose software and in specialized packages [29], as the development of modular and customizable open-source software for surface analysis is an emerging trend. Surface roughness measurements are conducted using various instruments based on both contact and non-contact techniques [30,31], employing either profile-based or areal (surface) measurement methods. The comparison between these approaches has been extensively discussed in the literature for many years [32,33,34]. Contact profilometers operate by dragging a diamond-tipped stylus across the surface [35,36], whereas optical profilometers utilize confocal and interferometric principles. Surface data acquisition equipment includes coherent scanning interferometers [37,38], confocal microscopes [39,40], and focus variation microscopes [41,42]. For capturing finer surface features, techniques based on the atomic properties of materials are employed—such as scanning and transmission electron microscopy (SEM and TEM), scanning tunneling microscopy (STM), and atomic force microscopy (AFM). Recently, efforts have also been made to apply computed tomography (CT) for surface characterization, which appears to be a promising direction [43,44]. Unlike conventional surface measurement methods, CT enables the evaluation of microtopography on complex, curved, or partially inaccessible surfaces without physical contact or surface alteration. Especially in applications involving rougher surfaces where fast measurements are required, laser and other line triangulation sensors are increasingly used [45,46,47]. The accuracy of a given surface measurement depends on several factors, including the measurement method itself, the precision of mechanical and optical components, system resolution, and the measurement and environmental conditions.

Innovations are also emerging in the way surfaces are described and analyzed. Some modern measurement systems—such as computed tomography scanners—enable the acquisition of surfaces with complex geometries, for which current definitions of texture parameters may be insufficient. The characterization of such surfaces, particularly those containing re-entrant features, requires a specialized and adapted approach [48]. Artificial intelligence (AI) is playing an increasingly important role in surface metrology [49], including the use of neural networks [50,51] and advanced image processing algorithms [52,53], and its relevance across various aspects of the measurement process is expected to grow in the coming years. This includes support for operators, especially in light of growing challenges related to the availability of highly skilled personnel.

Today, surface features at the microscale have become one of the key criteria in selecting manufacturing methods. Increasingly, micro- and nanostructural analysis is regarded as equally important as classical dimensional and geometric evaluation [54]. This subject is addressed in numerous studies across a range of production technologies, including both subtractive manufacturing processes [55] and additive manufacturing methods [56].

In engineering practice, a growing synergy can be observed between surface roughness metrology and tribology, as in both disciplines the surface—acting as the boundary interface with the environment—plays a critical role. The integration of surface metrology and tribology not only enables the assessment of the manufacturing quality of individual components and assemblies but also allows for the prediction of their behavior under dynamic conditions. This makes it a fundamental aspect of modern approaches to designing durable and efficient technical systems.

## 3. Topics Analyzed in Special Issues

Turning to the contents of the Special Issues titled “Surface Inspection and Description in Metrology and Tribology” (see List of Contributions below), in light of the above analysis, they address highly relevant topics at the intersection of contemporary science and industry. The first volume, discussed in detail in a previous editorial [57], covered surface description at the micro- (contribution 1 [58]), meso- (contribution 2 [59]), and macroscales (contribution 3 [60]), as well as issues related to machining processes (contributions 4 [61] and 5 [62]), abrasive treatments (contribution 6 [63]), and additive manufacturing (contribution 7 [64]). Tribological topics such as wear mechanisms (contributions 8 [65] and 9 [66]), coatings (contribution 10 [67]), and adhesion phenomena (contribution 11 [68]) were also featured. In addition, the field of biotribology was represented by a contribution focused on dental applications (contribution 12 [69]). It is worth noting that some of these publications have already garnered over 150 citations, underscoring their high impact. The second volume presents an equally rich collection of material, once again addressing both metrological and tribological challenges. The contributions reflect the ongoing interdisciplinary dialog and the growing importance of surface characterization and functionality in a wide range of scientific and engineering domains.

The fundamental components of quantitative surface characterization are parameters and functions. This topic was addressed in the article by Pawlus et al. (contribution 13 [70]). Surface topography, in general, is not easy to characterize due to the large variety of features it may exhibit. This task becomes even more complex in the case of textured, functionally relevant surfaces designed for tribological applications. As a result, for a basic description of random two-process surfaces, five parameters were sufficient, textured surfaces with isolated oil-retaining pockets required at least six, while for a broader scientific characterization—regardless of the surface type—a set of seven parameters provided the essential surface information. A related topic was addressed in contribution 14 [71], which focused on the characterization of the maximum height of a surface texture. Due to the high variability of the maximum profile height, a more stable parameter can be derived by averaging multiple parallel profile measurements extracted from surface topography data. Using this approach, it becomes possible to accurately estimate the maximum profile amplitude (Pt) and other profile parameters such as Pq (root mean square roughness), Pa (arithmetical mean height), and shape-related ratios like Pq/Pa and Pp/Pt, which describe the distribution of profile ordinates. The Pq/Pa and Pp/Pt ratios were found to be more stable than kurtosis (Pku) and skewness (Psk). However, surface texture measurements based on surface parameters are highly susceptible to measurement errors (contribution 15 [72]). Different types of errors affect texture parameters in various ways [73,74]. To investigate the impact of surface scratches, the researchers artificially introduced round valleys of varying diameters into surface textures measured using a white-light interferometer(Talysurf CCI Lite, Taylor Hobson, Leicester, England). The results showed that measurement errors increased significantly with the size of the scratches introduced, highlighting the sensitivity of certain parameters to surface damage.

The surface image derived from measurement data can be interpreted as a topographic surface map, the fluctuations of which were analyzed in contribution 16 [75]. The study presented and evaluated a top–down method for quantitatively determining height fluctuations on topographic maps, based directly on repeated surface measurements at fixed locations of interest. In this method, measured heights at each position are statistically analyzed across multiple consecutive measurements, performed without moving the measured object relative to the instrument. The findings showed that very few unmeasured points persist consistently across all measurements at any given location, and the uncertainty distributions resemble those of certain features on the topographic maps at the same sites. This suggests that topographic features themselves may amplify measurement fluctuations.

Concluding the discussion on surface characterization, the ability to objectively assess surface finish in order to ensure consistent visual appearance is a critical requirement in the field of surface coatings engineering. This was demonstrated in contribution 17 [76], which presented how computational frameworks—referred to as Surface Quality Inspection Descriptors (SQuID™)—can be used to effectively classify different surface finish appearance categories, particularly in the context of powder-coated surfaces. The study compared the automated classification based on multiscale surface texture parameters with results obtained from a manual gloss meter. The findings indicated that high-accuracy automated classification is feasible, representing a promising direction for consistent and efficient surface appearance evaluation.

Turning to material processing topics, Pereira et al. investigated the manufacturing and analysis of steel inclined walls produced using directed energy deposition-based techniques. The three-dimensional analysis of these components was the subject of contribution 18 [77], where the authors examined the geometry and surface topography of steel walls fabricated using wire arc additive manufacturing (WAAM) under various processing conditions and inclination angles, without any additional support structures except for the base substrate. It was found that travel speed played a significant role in determining the cross-sectional geometry, primarily due to the amount of heat input into the weld zone. This paper contributes to optimizing process parameters in the additive manufacturing of inclined geometries, improving the dimensional consistency and surface quality of WAAM-fabricated components.

Surfaces produced through non-material-removal (plastic deformation) processes were also addressed in this Special Issue. Swirad (contribution 19 [78]) investigated surface texture changes resulting from the ball burnishing process applied to steel surfaces previously milled or ground, and demonstrated that ball burnishing significantly reduced the surface roughness amplitude. The decrease in surface height parameters was more pronounced when the initial texture amplitude was higher, and hybrid parameters were also reduced following the treatment.

Hawryluk et al. (contribution 20 [79]) focused on the analysis of forged surfaces in die hot-forging processes. They presented industrial-scale results related to die forging, incorporating the use of advanced measurement techniques, numerical simulations, and digital tools and methods for geometric analysis and defect detection. The findings indicate that such tools and techniques hold significant potential for reducing measurement time and increasing accuracy, thereby supporting more efficient and precise process monitoring and quality control in forging operations. Staying within the context of forging, tool durability is another critical issue and was the focus of analysis presented in contribution 21 [80]. The study examined the influence of material microstructure and hardness on tool life, contributing to a better understanding of the underlying wear mechanisms. The researchers utilized tools subjected to nitriding treatments, as well as those coated with hybrid CrAlSiN and CrAlBN coatings, which were shown to significantly reduce tool wear and enhance service life. The authors also proposed a Z coefficient as a convenient metric for expressing material loss relative to the number of forged parts produced. Another research team investigated the deep drawing process, with a particular focus on the friction effects that hinder plastic deformation and degrade the surface quality of drawn components (contribution 22 [81]). To improve this situation, the authors proposed the use of lubricants containing hard solid particles. The experiments were conducted on dual-phase steel sheets, using sunflower and rapeseed oils with silicon dioxide (SiO_2_) particles added at varying pressure levels. The influence of friction on surface roughness changes of the steel sheets was also systematically evaluated.

Surface topography is also a critical factor following abrasive machining processes. Kacalak et al. (contribution 23 [82]) presented probabilistic aspects of grinding process diagnostics, emphasizing metrological aspects of assessing the topography of machined surfaces. The authors described the mechanisms of surface geometric structure formation in grinding, noting that the distribution of features relevant to process diagnostics depends on the cumulative effects of random disturbances. They proposed a method for determining the classification capability of specific parameters used to evaluate the stereometric properties of ground surfaces. Additionally, a methodology was introduced to assess the ability of various surface parameters to discriminate between different geometric structures, supporting more effective diagnostics of surface integrity and process stability. Lisowicz et al. (contribution 24 [83]), in turn, investigated tool wear in finish turning of Ti-6Al-4V titanium alloy. They employed recurrence plot (RP) analysis and recurrence quantification analysis (RQA) to monitor tool wear under minimum quantity lubrication (MQL) conditions. The results showed that recurrence-based analysis can effectively track the dynamics of the cutting process, offering a promising tool for real-time wear monitoring and process control.

Wear is also a critical factor in operational performance and durability. One example is the issue of water–sand flow, for which an erosion–corrosion failure analysis was presented in contribution 25 [84]. The authors investigated leakage in a pipe nozzle using various surface analysis techniques and conducted numerical simulations based on computational fluid dynamics (CFD) combined with the discrete phase model (DPM). The results revealed that increased turbulence and repeated particle impacts on the inner wall led to the development of leakage points and cracks in the pipe nozzle. As a mitigation strategy, the authors proposed a design modification to the reducer section, which effectively reduced erosion-induced wear. Another example of service-related research involved the assessment of the durability and reliability of organic coatings applied to exterior surfaces of roofing steel sheets (contribution 26 [85]). The coatings’ longevity was evaluated by examining their resistance to tribological wear. The tests confirmed that the structure of the coating plays a key role in determining the durability and functional reliability of the final product.

As seen in the List of Contributions, bioengineering also plays a vital role in this collection. The article titled “The Influence of Osteon Orientation on Surface Topography Parameters after Machining of Cortical Bone Tissue” (contribution 27 [86]) explored this domain in depth. Mechanical processing of cortical bone is among the most common surgical procedures, and one of the critical aspects associated with it is the condition of the surface layer, which can promote tissue regeneration and serve as a drug delivery platform. The study involved a comparative analysis of surface conditions before and after orthogonal and abrasive machining, aiming at confirming the influence of both the machining mechanism and the orthotropic properties of bone tissue on the resulting surface topography. The findings suggest that cutting in directions both transverse and parallel to the osteon axis is preferable, due to the favorable properties of the developed bone surface in those orientations.

## 4. Conclusions

The above discussion clearly demonstrates the importance of surface roughness evaluation in modern science and engineering. Three-dimensional surface topography encompasses not only roughness (interpreted as short-wavelength irregularities) but also waviness (longer-wavelength surface deviations) and form at the meso- or macroscale. Every measured surface exhibits features across macro-, meso-, and micro-/nanoscales. Height variations under mechanical loading lead to localized stress concentrations, making surface topography a critical factor in determining the fatigue resistance of the surface layer. It has been shown that high peaks and deep valleys weaken fatigue strength to a greater extent than what might be inferred from average roughness parameters alone. Moreover, solid surfaces possess a certain amount of surface energy, which results from unbalanced intermolecular forces at phase boundaries. A high surface free energy increases the tendency toward adhesive friction, promoting material wear through fragment removal during bond rupture. Thus, the surface is not merely a boundary of a solid body but rather an active functional zone, whose properties can be deliberately engineered at the micro- and nanometric scale. The interdisciplinary nature of surface engineering research—integrating aspects of solid-state physics, chemistry, mechanics, materials technology, and nanotechnology—makes it one of the pillars of contemporary high-tech innovation, and a field with substantial transformative potential across modern manufacturing and product design.

## Data Availability

Data sharing is not applicable to this article.
